# Supplementary material for: A robust gene-stacking method utilizing yeast assembly for plant synthetic biology
Source: Nat Commun. 2016 Oct 26;7:13215. doi: 10.1038/ncomms13215 (PMC5095168; doi:10.1038/ncomms13215)
Supplement: Supplementary Information — Supplementary Figures 1-3, Supplementary Tables 1-7 and Supplementary References [file ncomms13215-s1.pdf]

Supplemental Figures:

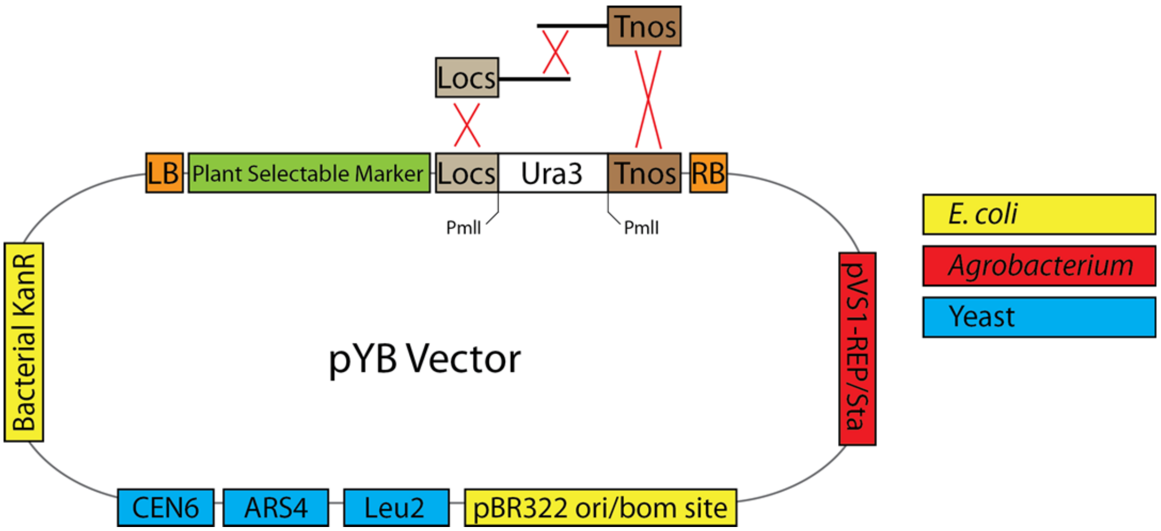

Supplementary Figure 1. General schematic of yeast assembly into pYB vector backbones

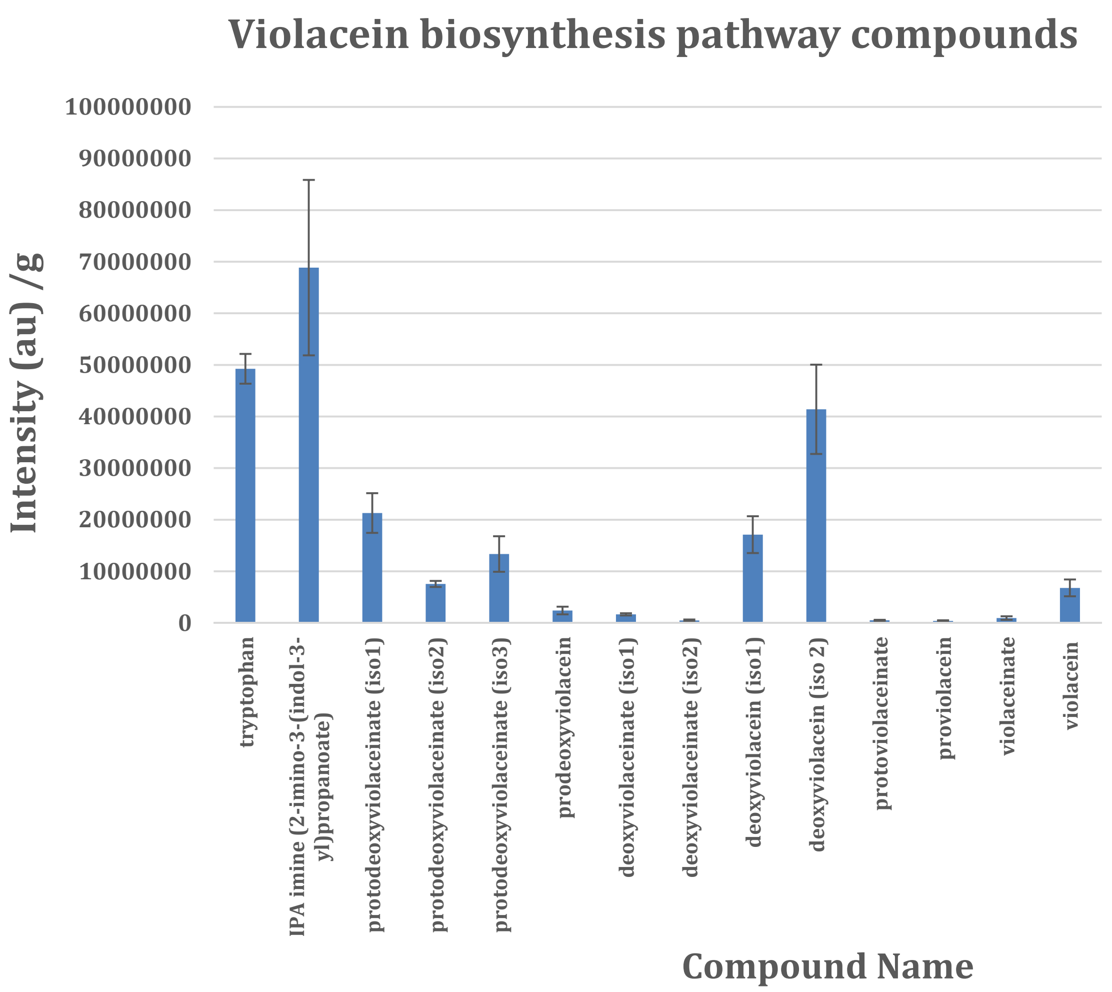

Supplementary Figure 2. Relative intensity of violacein biosynthesis compounds and intermediates detected by LC-MS. (iso = isomer). Error bars indicate standard deviation, n = 3.

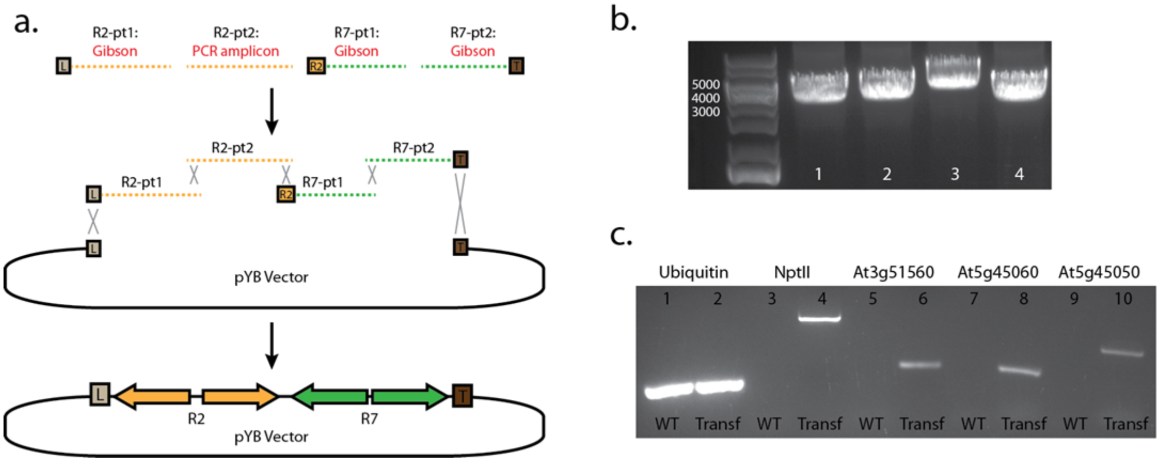

Supplementary Figure 3. Mixed jStack assembly strategy of a synthetic gene. cluster. a, Fusing the R2 and R7 gene cluster together via yeast assembly demonstrates the versatility of the jStack platform, where a combination of Gibson assembly and direct PCR amplicons can be mixed together to assemble via yeast homologous recombination to generate synthetic gene clusters. The linker (L) and terminator (T) homology arms that allow for recombination into the pYB vector is Gibson assembled to PCR amplicons of a given gene cluster fragment. The 3’end of the R2 gene cluster is Gibson assembled to the beginning of the R7 gene cluster fragment, enabling recombination between the two gene clusters. Ultimately, the four fragments are yeast assembled together into a pYB vector. **b,** PCR using the assembled pYB vector as template, confirming assembly of the four R genes from the two fused gene clusters stacked into one vector. PCR amplicons across the whole coding sequences for At3g51560 from gene cluster R2 (Lane 1, expected size 4106 bp), At3g51570 from gene cluster R2 (Lane 2, expected size 4099 bp), At5g45050 from gene cluster R7 (Lane 3, expected size 4790 bp), At5g45060 from gene cluster R7 (Lane 4, expected size 4030 bp). **c,** RT-PCR was conducted on wild type and stably transformed roots of soybean via *Agrobacterium rhizogenes*, demonstrating expression of genes within the synthetic gene cluster in a heterologous host. Positive control PCRs using ubiquitin (Glyma20g27950) are shown in lanes 1 (wild type) and 2 (transformed roots) with an expected size of 162 bp. The transformed root expressed the neomycin phosphotransferase gene (lane 4, expected size 623 bp) R genes At3g51560 (lane 6, expected size 240 bp), At5g45060 (lane 8, expected size 239 bp), and At5g45050 (lane 10, expected size 371 bp), whereas no band was observed in the wild type roots (lane 3, 5, 7, and 9 respectively).

Supplemental Tables:

Supplementary Table 1. List of plasmid backbones generated for jStack assembly

Name	Type of Plasmid	Description	E. coli ori	E. coli selection	Strain Part ID
pPMS008	Level 1 Intermediary Vector	Level 1 gene cassette assembly	ColE1 (high copy)	Chloramphenicol	JBx_049714
pPMS028	Level 1 Intermediary Vector	Level 1 gene cassette assembly	p15A (low-medium copy)	Chloramphenicol	JBx_049716
pYB1301	Level 2 pYB Vector	pCAMBIA1301 derivative, plant hygromycin selectable marker	pBR322 ori (low-medium copy)	Kanamycin	JBx_049708
pYB2301	Level 2 pYB Vector	pCAMBIA2301 derivative, plant kanamycin selectable marker	pBR322 ori (low-medium copy)	Kanamycin	JBx_049710
pYB3301	Level 2 pYB Vector	pCAMBIA3301 derivative, plant Basta selectable marker	pBR322 ori (low-medium copy)	Kanamycin	JBx_049712

Supplementary Table 2. Library of plant-specific Level 0 DNA parts

Plasmid Name	Part Name	Part Type	Origin	Description	Strain Part ID	Reference
pms5055	{L_tOcs}	Linker	*Agrobacterium*	Octopine Synthase, primary linker	JBx_042264	used in Farhi et al, Nat Biotech 2011[1]
pms5056	{T_tNos}	Terminator	*Agrobacterium*	Nopaline Synthase terminator	JBx_042266	used in Farhi et al, Nat Biotech 2011[1]
pms5057	{P_AtFBA2}	Promoter	*Arabidopsis*	Fructose-bisphosphate aldolase 2 promoter	JBx_042268	Lu et al, Gene 2012[2]
pms5058	{P_AtRbcs1A}	Promoter	*Arabidopsis*	Rubisco Small Subunit promoter	JBx_042270	used in Farhi et al, Nat Biotech 2011[1]
pms5059	{P_AtC4H}	Promoter	*Arabidopsis*	Cinnamate-4-hydroxylase, secondary cell wall promoter	JBx_042272	used in Yang F et al, Plant Biotech J 2013[3]
pms5060	{P_AtC4H_short}	Promoter	*Arabidopsis*	Cinnamate-4-hydroxylase, secondary cell wall promoter (truncated)	JBx_042274	Yang F et al, Plant Biotech J 2013[3]
pms5062	{P_nos}	Promoter	*Agrobacterium*	Nopaline synthase promoter	JBx_042276	used in Farhi et al, Nat Biotech 2011[1]
pms5063	{P_MAS}	Promoter	*Agrobacterium*	Mannopine synthase promoter	JBx_042278	used in Langridge et al, PNAS 1989[4]
pms5064	{T_tAtHsp}	Terminator	*Arabidopsis*	Heat shock protein terminator	JBx_042280	promoter used in Farhi et al, Nat Biotech 2011[1]
pms5065	{T_tAtRbcS}	Terminator	*Arabidopsis*	Rubisco Small Subunit terminator	JBx_042282	used in Farhi et al, Nat Biotech 2011[1]
pms5066	{T_MAS}	Terminator	*Arabidopsis*	Mannopine synthase terminator	JBx_042284	promoter used in Langridge et al, PNAS 1989[4]
pms5067	{L_tAtHsp}	Linker	*Arabidopsis*	Heat shock protein linker	JBx_042286	used in Sarrion-Perdigones et al, Plant Physiol 2013[5]
pms5068	{L_tAtRbcS}	Linker	*Arabidopsis*	Rubisco Small Subunit linker	JBx_042288	used in Farhi et al, Nat Biotech 2011[1]
pms5069	{L_MAS}	Linker	*Agrobacterium*	Mannopine synthase linker	JBx_042290	promoter used in Langridge et al, PNAS 1989[4]
pms5070	{C_GFP}	CDS	synthesized	GFP	JBx_042292	Lao et al, Plant J 2014[6]
pms5071	{C_GFP_attEpitope}	CDS	synthesized	GFP-attEpitope	JBx_042294	Lao et al, Plant J 2014[6]
pms5072	{C_sfGFP}	CDS	synthesized	superfolder GFP	JBx_042296	Kittleson et al, J Biol Eng 2011[7]
pms5077	{P_AtUbq10}	Promoter	*Arabidopsis*	Polyubiquitin 10 promoter	JBx_042298	used in Sarrion-Perdigones et al, Plant Physiol 2013[5]
pms5078	{P_AtHsp18}	Promoter	*Arabidopsis*	Heat shock protein 18 promoter	JBx_042300	used in Farhi et al, Nat Biotech 2011[1]
pms5079	{P_AtHsp70}	Promoter	*Arabidopsis*	Heat shock protein 70 promoter	JBx_042302	used in Sung et al, Plant Physiol 2001[8]
pms5080	{P_AtAct2}	Promoter	*Arabidopsis*	Actin 2 promoter	JBx_042304	used in Sarrion-Perdigones et al, Plant Physiol 2013[5]
pms5081	{P_At2S3}	Promoter	*Arabidopsis*	AT2S3 seed specific promoter	JBx_042306	used in Emami et al, Front Plant Sci 2013[9]
pms5082	{P_AtBch1}	Promoter	*Arabidopsis*	Beta carotenoid hydroxylase 1 promoter	JBx_042308	used in Sarrion-Perdigones et al, Plant Physiol 2013[5]
pms5083	{P_AtUbq3}	Promoter	*Arabidopsis*	Ubiquitin 3 promoter	JBx_042310	used in Sarrion-Perdigones et al, Plant Physiol 2013[5]
pms5084	{P_AtPYK10}	Promoter	*Arabidopsis*	PYK10 root specific promoter	JBx_042312	used in Nitz et al, Plant Sci 2001[10]
pms5085	{P_AtJAL34}	Promoter	*Arabidopsis*	JAL34 predicted root specific promoter	JBx_042314	Yamada et al, Plant Cell Physiol 2011[11]
pms5086	{P_AtWRKY6}	Promoter	*Arabidopsis*	WRKY6 predicted root specific promoter	JBx_042316	Robatzek and Somssich, Genes Dev 2002[12]
pms5087	{P_At3g24240}	Promoter	*Arabidopsis*	root specific promoter	JBx_042318	used in Shinohara et al, PNAS, 2016[13]
pms5088	{P_At2g45430}	Promoter	*Arabidopsis*	predicted root specific promoter	JBx_042320	Zhao et al, PNAS 2013[14]
pms5089	{P_AtHY5}	Promoter	*Arabidopsis*	Elongated Hypocotyl 5 promoter	JBx_042322	Chattopadhyay et al, Plant Cell 1998[15]
pms5090	{T_AtAct2}	Terminator	*Arabidopsis*	Actin 2 terminator	JBx_042324	used in Sarrion-Perdigones et al, Plant Physiol 2013[5]

pms5091	{T_AtBch1}	Terminator	*Arabidopsis*	Beta carotenoid hydroxylase 1 terminator	JBx_042326	used in Sarrion-Perdigones et al, Plant Physiol 2013[5]
pms5092	{T_AtHsp18.2}	Terminator	*Arabidopsis*	Heat shock protein 18 terminator	JBx_042328	promoter used in Farhi et al, Nat Biotech 2011[1]
pms5093	{T_AtUbq3}	Terminator	*Arabidopsis*	Ubiquitin 3 terminator	JBx_042330	used in Sarrion-Perdigones et al, Plant Physiol 2013[5]
pms5094	{T_AtPYK10}	Terminator	*Arabidopsis*	PYK10 terminator	JBx_042332	promoter characterized in Nitz et al, Plant Sci 2001[10]
pms5095	{T_AtJAL34}	Terminator	*Arabidopsis*	JAL34 terminator	JBx_042334	gene characterized in Yamada et al, Plant Cell Physiol 2011[37]
pms5096	{T_AtWRKY6}	Terminator	*Arabidopsis*	WRKY6 terminator	JBx_042336	promoter characterized in Robatzek and Somssich, Genes Dev 2002[38]
pms5097	{T_At3g24240}	Terminator	*Arabidopsis*	At3g24240 terminator	JBx_042338	promoter used in Shinohara et al, PNAS, 2016[39]
pms5098	{T_At2g45430}	Terminator	*Arabidopsis*	At2g45430 terminator	JBx_042340	Zhao et al, PNAS 2013[40]
pms5099	{T_AtHY5}	Terminator	*Arabidopsis*	Elongated Hypocotyl 5 terminator	JBx_042342	Chattopadhyay et al, Plant Cell 1998[15]
pms5100	{L_AtAct2}	Linker	*Arabidopsis*	Actin 2 linker	JBx_042344	used in Sarrion-Perdigones et al, Plant Physiol 2013[5]
pms5101	{L_AtBch1}	Linker	*Arabidopsis*	Beta carotenoid hydroxylase 1 linker	JBx_042346	used in Sarrion-Perdigones et al, Plant Physiol 2013[5]
pms5102	{L_AtHsp18.2}	Linker	*Arabidopsis*	Heat shock protein 18 linker	JBx_042348	promoter used in Farhi et al, Nat Biotech 2011[1]
pms5103	{L_AtUbq3}	Linker	*Arabidopsis*	Ubiquitin 3 linker	JBx_042350	used in Sarrion-Perdigones et al, Plant Physiol 2013[5]
pms5104	{L_AtPYK10}	Linker	*Arabidopsis*	PYK10 linker	JBx_042352	promoter used in Nitz et al, Plant Sci 2001[10]
pms5105	{L_AtJAL34}	Linker	*Arabidopsis*	JAL34 linker	JBx_042354	Yamada et al, Plant Cell Physiol 2011[11]
pms5106	{L_AtWRKY6}	Linker	*Arabidopsis*	WRKY6 linker	JBx_042356	Robatzek and Somssich, Genes Dev 2002[12]
pms5107	{L_At3g24240}	Linker	*Arabidopsis*	At3g24240 linker	JBx_042358	promoter used in Shinohara et al, PNAS, 2016[13]
pms5108	{L_At2g45430}	Linker	*Arabidopsis*	At2g45430 linker	JBx_042360	Zhao et al, PNAS 2013[14]
pms5109	{L_AtHY5}	Linker	*Arabidopsis*	Elongated Hypocotyl 5 linker	JBx_042362	Chattopadhyay et al, Plant Cell 1998[15]
pms5125	{P_Casp1}	Promoter	*Arabidopsis*	Casp1 root endodermal promoter	JBx_042364	used in Naseer et al, PNAS 2012[16]
pms5126	{P_DAISY}	Promoter	*Arabidopsis*	DAISY root endodermal promoter	JBx_042366	used in Naseer et al, PNAS 2012[16]
pms5127	{P_KCR1}	Promoter	*Arabidopsis*	KCR1 root endodermal promoter	JBx_042368	used in Naseer et al, PNAS 2012[16]
pms5128	{P_HORST}	Promoter	*Arabidopsis*	HORST root endodermal promoter	JBx_042370	used in Naseer et al, PNAS 2012[16]
pms5129	{P_CYP86B1}	Promoter	*Arabidopsis*	CYP86B1 root endodermal promoter	JBx_042372	used in Naseer et al, PNAS 2012[16]
pms5130	{P_CYP705A1}	Promoter	*Arabidopsis*	CYP705A1 root endodermal promoter	JBx_042374	used in Naseer et al, PNAS 2012[16]
pms5131	{P_GPAT5}	Promoter	*Arabidopsis*	GPAT5 root endodermal promoter	JBx_042376	used in Naseer et al, PNAS 2012[16]
pms5132	{P_ASFT}	Promoter	*Arabidopsis*	ASFT root endodermal promoter	JBx_042378	used in Naseer et al, PNAS 2012[16]
pms5133	{P_FAR4}	Promoter	*Arabidopsis*	FAR4 root endodermal promoter	JBx_042380	used in Naseer et al, PNAS 2012[16]
pms5206	{C_synVioA}	CDS	synthesized	VioA, violacein biosynthesis enzyme	JBx_042382	characterized in this study
pms5207	{C_synVioB}	CDS	synthesized	VioB, violacein biosynthesis enzyme	JBx_042384	characterized in this study
pms5208	{C_synVioC}	CDS	synthesized	VioC, violacein biosynthesis enzyme	JBx_042386	characterized in this study
pms5209	{C_synVioD}	CDS	synthesized	VioD, violacein biosynthesis enzyme	JBx_042388	characterized in this study

pms5210	{C_synVioE}	CDS	synthesized	VioE, violacein biosynthesis enzyme	JBx_042390	characterized in this study
pms5793	{T_tG7}	Terminator	*Agrobacterium*	tG7 terminator	JBx_042392	used in Butaye et al, Plant J 2004[17]
pms5794	{L_tG7}	Linker	*Agrobacterium*	tG7 linker	JBx_042394	used in Butaye et al, Plant J 2004[17]
pms5124	{C_dsRed}	CDS	synthesized	dsRed	JBx_062441	Lao et al, Plant J 2014[6]
pms5460	{C_GUS}	CDS	synthesized	β-glucuronidase	JBx_062443	Wally et al, Plant Cell Rep 2008[18]
pms5792	{P_SlE8}	Promoter	Tomato	SlE8 fruit specific promoter	JBx_062445	used in Butelli et al, Nat Biotech 2008[19]
pms5841	{P_OsHPX1}	Promoter	Rice	HPX1 root specific promoter	JBx_062447	used in Park et al, Plant Biotech Rep 2013[20]
pms5842	{P_TobRB7}	Promoter	Tobacco	TobRB7 root specific promoter	JBx_062449	used in Yamamoto et al, Plant Cell 1991[21]
pms5878	{P_ZmUBQ1}	Promoter	*Maize*	Ubiquitin 1 promoter	JBx_062451	used in Cornejo et al, Plant Mol Biol 1993[22]
pms5916	{C_synBisSyn}	CDS	synthesized	Bisabolene synthase	JBx_062732	characterized in this study
pms5917	{C_c1-synBisSyn}	CDS	synthesized	Chloroplast targetted bisabolene synthase	JBx_062734	characterized in this study
pms5925	{C_c2-EcFPPS}	CDS	synthesized	E. coli FPP synthase	JBx_062736	characterized in this study
pms5928	{C_c3-EcDXS}	CDS	synthesized	E. coli 1-Deoxy-D-xylulose 5-phosphate synthase	JBx_062459	characterized in this study
pms6116	{P_AtIrx5}	Promoter	*Arabidopsis*	Irx5 secondary cell wall promoter	JBx_062461	used in Gondolf et al, BMC Plant Biol 2014[23]
pms6117	{P_AtIrx8}	Promoter	*Arabidopsis*	Irx8 secondary cell wall promoter	JBx_062463	Yang et al, Plant Biotech J 2013[23]
pms6126	{P_AtCesA7}	Promoter	*Arabidopsis*	CesA7 secondary cell wall promoter	JBx_062465	used in Kim et al, Plant J 2013[24]
pms6392	{P_AtNST1}	Promoter	*Arabidopsis*	NST1 secondary cell wall promoter	JBx_062467	used in Vega-Sanchez et al, Plant Biotech J 2015[25]
pms6393	{P_AtSAG12}	Promoter	*Arabidopsis*	SAG12 senescence promoter	JBx_062469	used in Vega-Sanchez et al, Plant Biotech J 2015[26]
pms6394	{P_AtVND6}	Promoter	*Arabidopsis*	VND6 secondary cell wall promoter	JBx_062471	used in Kubo et al, Genes and Dev 2005[26]
pms6395	{P_AtVND7}	Promoter	*Arabidopsis*	VND7 secondary cell wall promoter	JBx_062473	used in Kubo et al, Genes and Dev 2005[26]
pms6396	{T_AtNST1}	Terminator	*Arabidopsis*	NST1 terminator	JBx_062475	promoter used in Vega-Sanchez et al, Plant Biotech J 2015[25]
pms6397	{T_AtSAG12}	Terminator	*Arabidopsis*	SAG12 terminator	JBx_062477	promoter used in Vega-Sanchez et al, Plant Biotech J 2015[25]
pms6398	{T_AtVND6}	Terminator	*Arabidopsis*	VND6 terminator	JBx_062479	promoter used in Kubo et al, Genes and Dev 2005[26]
pms6399	{T_AtVND7}	Terminator	*Arabidopsis*	VND7 terminator	JBx_062481	promoter used in Kubo et al, Genes and Dev 2005[26]
pms6400	{L_AtNST1}	Linker	*Arabidopsis*	NST1 linker	JBx_062483	promoter used in Vega-Sanchez et al, Plant Biotech J 2015[25]
pms6401	{L_AtSAG12}	Linker	*Arabidopsis*	SAG12 linker	JBx_062485	promoter used in Vega-Sanchez et al, Plant Biotech J 2015[25]
pms6402	{L_AtVND6}	Linker	*Arabidopsis*	VND6 linker	JBx_062487	promoter used in Kubo et al, Genes and Dev 2005[26]
pms6403	{L_AtVND7}	Linker	*Arabidopsis*	VND7 linker	JBx_062489	promoter used in Kubo et al, Genes and Dev 2005[26]
pms6404	{T_AtHsp70}	Terminator	*Arabidopsis*	Hsp70 terminator	JBx_062491	Sung et al, Plant Physiol 2001[8]
pms6405	{T_At2S3}	Terminator	*Arabidopsis*	At2S3 terminator	JBx_062493	promoter characterized in Emami et al, Front Plant Sci 2013[9]
pms6406	{T_AtFBA2}	Terminator	*Arabidopsis*	FBA2 terminator	JBx_062495	Lu et al, Gene 2012[2]
pms6408	{T_AtCASP1}	Terminator	*Arabidopsis*	CASP1 terminator	JBx_062497	promoter characterized in Naseer et al, PNAS 2012[16]
pms6409	{T_AtKCR1}	Terminator	*Arabidopsis*	KCR1 terminator	JBx_062499	promoter characterized in Naseer et al, PNAS 2012[16]
pms6410	{T_AtASFT}	Terminator	*Arabidopsis*	ASFT terminator	JBx_062501	promoter characterized in Naseer et al, PNAS 2012[16]
pms6411	{L_AtHsp70}	Linker	*Arabidopsis*	Hsp70 linker	JBx_062503	Sung et al, Plant Physiol 2001[8]

pms6412	{L_At2S3}	Linker	*Arabidopsis*	At2S3 linker	JBx_062505	promoter characterized in Emami et al, Front Plant Sci 2013[9]
pms6413	{L_AtFBA2}	Linker	*Arabidopsis*	FBA2 linker	JBx_062507	Lu et al, Gene 2012[2]
pms6414	{L_AtUbq10}	Linker	*Arabidopsis*	Ubq10 linker	JBx_062509	promoter used in Sarrion-Perdigones et al, Plant Physiol 2013[5]
pms6416	{L_AtCASP1}	Linker	*Arabidopsis*	CASP1 linker	JBx_062511	promoter characterized in Naseer et al, PNAS 2012[16]
pms6417	{L_AtKCR1}	Linker	*Arabidopsis*	KCR1 linker	JBx_062513	promoter characterized in Naseer et al, PNAS 2012[16]
pms6418	{L_AtASFT}	Linker	*Arabidopsis*	ASFT linker	JBx_062515	promoter characterized in Naseer et al, PNAS 2012[16]
pms6422	{P_AtAMT1.1}	Promoter	*Arabidopsis*	AMT1.1 promoter	JBx_062517	used in Loque et al, Plant J 2006[27]
pms6424	{P_AtPht1.1}	Promoter	*Arabidopsis*	Pht1.1 promoter	JBx_062519	used in Mudge et al, Plant J 2002[28]
pms6425	{P_AtPht1.4}	Promoter	*Arabidopsis*	Pht1.4 ptomoter	JBx_062521	used in Mudge et al, Plant J 2002[28]
pms6426	{P_AtIRT1.1}	Promoter	*Arabidopsis*	IRT1.1 promoter	JBx_062523	used in Vert et al, Plant Cell 2002[29]
pms6433	{T_AtIRT1.2}	Terminator	*Arabidopsis*	IRT1.2 terminator	JBx_062525	Varotto et al, Plant J 2002[30]
pms6434	{L_AtAMT1.1}	Linker	*Arabidopsis*	AMT1.1 linker	JBx_062527	promoter used in Loque et al, Plant J 2006[27]
pms6435	{L_AtAMT1.3}	Linker	*Arabidopsis*	AMT1.3 linker	JBx_062529	promoter used in Loque et al, Plant J 2006[273]
pms6436	{L_AtPht1.1}	Linker	*Arabidopsis*	Pht1.1 linker	JBx_062531	promoter used in Mudge et al, Plant J 2002[28]
pms6437	{L_AtPht1.4}	Linker	*Arabidopsis*	Pht1.4 linker	JBx_062533	promoter used in Mudge et al, Plant J 2002[28]
pms6438	{L_AtIRT1.1}	Linker	*Arabidopsis*	IRT1.1 linker	JBx_062535	promoter used in Vert et al, Plant Cell 2002[29]
pms6439	{L_AtIRT1.2}	Linker	*Arabidopsis*	IRT1.2 linker	JBx_062537	Varotto et al, Plant J 2002[30]

Supplementary Table 3. jStack-assembled constructs used in this study

Construct name	Level	Description	Assembled Parts
**Reporter construct**			
**pms5463**	Level 2	{L_tOcs}{P_AtUbq10}{C_GUS}{T_tAtRbcS}{P_nos}{C_GFP_attEpitope}{T_AtUbq3}{P_MAS}{C_dsRed}{T_tNos}	pms5461 pms5462 pms5424
pms5461	Level 1	{L_tOcs}{P_AtUbq10}{C_GUS}{T_tAtRbcS}	pms5055 pms5077 pms5460 pms5065
pms5462	Level 1	{L_tAtRbcS}{P_nos}{C_GFP_attEpitope}{T_AtUbq3}	pms5068 pms5062 pms5071 pms5093
pms5424	Level 1	{L_AtUbq3}{P_MAS}{C_dsRed}{T_tNos}	pms5103 pms5063 pms5124 pms5056
**Bisabolene production**			
**pms6092**	Level 2	{L_tOcs}{P_nos}{C_synBisSyn}{T_tNos}	pms5055 pms5062 pms5916 pms5056
**pms6113**	Level 2	{L_tOcs}{P_MAS}{C_c2-EcFPPS}{T_tG7}{P_nos}{C_c1-synBisSyn}{T_tNos}	pms6105 pms6097
pms6105	Level 1	{L_tOcs}{P_MAS}{C_c2-EcFPPS}{T_tG7}	pms5055 pms5063 pms5925 pms5793
pms6097	Level 1	{L_tG7}{P_nos}{C_c1-synBisSyn}{T_tNos}	pms5794 pms5062 pms5917 pms5056
**pms6292**	Level 2	{L_tOcs}{P_AtRbcs1A}{C_c3-EcDXS}{T_tAtRbcS}{P_MAS}{C_c2-EcFPPS}{T_tG7}{P_nos}{C_c1-synBisSyn}{T_tNos}	pms6289 pms6288 pms6097
pms6289	Level 1	{L_tOcs}{P_AtRbcs1A}{C_c3-EcDXS}{T_tAtRbcS}	pms5055 pms5058 pms5928 pms5065
pms6288	Level 1	{L_tAtRbcS}{P_MAS}{C_c2-EcFPPS}{T_tG7}	pms5068 pms5063 pms5925 pms5793
pms6097	Level 1	{L_tG7}{P_nos}{C_c1-synBisSyn}{T_tNos}	pms5794 pms5062 pms5917 pms5056
**Violacein production**			
**pms5343**	Level 2	{L_tOcs}{P_AtHsp18}{C_synVioA}{T_tAtHsp}{P_MAS}{C_synVioB}{T_tAtRbcS}{P_AtAct2}{C_synVioE}{T_AtAct2}{P_AtRbcs1A}{C_synVioC}{T_AtUbq3}{P_AtBch1}{C_synVioD}{T_tNos}	pms5217 pms5218 pms5219 pms5220 pms5221
pms5217	Level 1	{L_tOcs}{P_AtUbq10}{C_synVioA}{T_tAtHsp}	pms5055 pms5077 pms5206 pms5064
pms5218	Level 1	{L_tAtHsp}{P_MAS}{C_synVioB}{T_tAtRbcS}	pms5067 pms5063 pms5207 pms5065
pms5219	Level 1	{L_tAtRbcS}{P_AtAct2}{C_synVioE}{T_AtAct2}	pms5068 pms5080 pms5210 pms5090
pms5220	Level 1	{L_AtAct2}{P_AtRbcs1A}{C_synVioC}{T_AtUbq3}	pms5100 pms5058 pms5208 pms5093
pms5221	Level 1	{L_AtUbq3}{P_AtBch1}{C_synVioD}{T_tNos}	pms5103 pms5082 pms5209 pms5056

Supplementary Table 4. Table of detected compounds by LC-MS

Compound Name	Formula	Neutral Mass	M+H (theoretical)	M+H (detected)	*m/z* difference (ppm)	Retention time, peak (min)
**tryptophan**	C11H12N2O2	204.0899	205.0972	205.0970	0.733	2.36
**IPA imine (2-imino-3-(indol-3-yl)propanoate)**	C11H9N2O2	202.0742	203.0815	203.0814	0.542	3.05
**protodeoxyviolaceinate (iso1)**	C21H14N3O2	341.1164	342.1237	342.1237	0.002	4.11
**protodeoxyviolaceinate (iso2)**	C21H14N3O2	341.1164	342.1237	342.1237	0.002	4.59
**protodeoxyviolaceinate (iso3)**	C21H14N3O2	341.1164	342.1237	342.1237	0.002	4.67
**prodeoxyviolacein**	C20H13N3O	311.1059	312.1131	312.1131	0.133	4.05
**deoxyviolaceinate (iso1)**	C21H14N3O3	357.1113	358.1186	358.1186	0.012	4.17
**deoxyviolaceinate (iso2)**	C21H14N3O3	357.1113	358.1186	358.1185	0.291	4.28
**deoxyviolacein (iso1)**	C20H13N3O2	327.1008	328.1080	328.1078	0.747	4.80
**deoxyviolacein (iso 2)**	C20H13N3O2	327.1008	328.1080	328.1080	0.137	4.66
**protoviolaceinate**	C21H14N3O3	357.1113	358.1186	358.1184	0.570	3.88
**proviolacein**	C20H13N3O2	327.1008	328.1080	328.1079	0.442	3.68
**violaceinate**	C21H14N3O4	373.1063	374.1135	374.1137	0.432	3.71
**violacein (sample)**	C20H13N3O3	343.0957	344.1030	344.1029	0.230	4.30
**violacein (standard)**	C20H13N3O3	343.0957	344.1030	344.1029	0.230	4.30
**chromopyrrolate (iso1)**	C22H13N3O4	385.1063	386.1135	386.1137	0.419	3.60
**chromopyrrolate (iso2)**	C22H13N3O4	385.1063	386.1135	386.1137	0.419	3.80
**chromopyrrolate (iso3)**	C22H13N3O4	385.1063	386.1135	386.1136	0.160	3.96
**chromopyrrolate (iso4)**	C22H13N3O4	385.1063	386.1135	386.1134	0.358	4.18

Supplementary Table 5. List of R gene clusters assembled

Name	R gene cluster	Cluster Size (bp)
R1	At2g17060/At2g17050	18,009
R2	At3g51570/At3g51560	10,623
R3	At4g12010/At4g12020	13,908
R4	At4g19530/At4g19520	19,127
R5	At4g36150/At4g36140	11,740
R6	At5g17880/At5g17890	15,965
R7	At5g45060/At5g45050	10,956
R8	At5g45200/At5g45210	16,440
R9	At5g45250/At5g45260 (RPS4/RRS1)	11,521
synthetic R2-R7	At3g51570/At3g51560/At5g45060/At5g45050	20,596

Supplementary Table 6. Primers used to generate library of plant-specific Level 0 DNA parts

Plasmid Name	Part Name	F primer name	R primer name	F primer sequence	R primer sequence
pms5055	{L_tOcs}	PMS175	PMS176	atatGAATTCggtctcagtccGAGGGGATCGAGCCCC	atatGGATCCggtctcactccCTGCTTTAATGAGATATGCGAGAC
pms5056	{T_tNos}	PMS177	PMS178	atatGAATTCggtctcagcttCGTTCAAACATTTGGCAATAAAGTTTC	atatGGATCCggtctcaagcgCCCGATCTAGTAACATAGATGACAC
pms5057	{P_AtFBA2}	PMS179	PMS180	atatAGAATTCcgtctcaggagCCTACACATCCACTTGGCC	atatAGATCTcgtctcacattTTTTTTTGTGTGTGTTATCTCTTTTCTC
pms5058	{P_AtRbcs1A}	PMS181	PMS182	atatAGAATTCcgtctcaggagAACATTCATACGTTTTCATTTGAAATATCC	atatGGATCCcgtctcacattTGTTCTTCTTTACTCTTTGTGTGAC
pms5059	{P_AtC4H}	PMS183	PMS184	atatGAATTCggtctcaggagCGGAATGAGAGACGAGAGC	atatGGATCCggtctcacattCCTAGGCGAGAGTAATTGAAAGC
pms5060	{P_AtC4H_short}	PMS185	PMS186	atatGAATTCggtctcaggagACAAACGTAATGCACTGTCG	atatGGATCCggtctcacattCCTAGGCGAGAGTAATTGAAAGC
pms5062	{P_nos}	PMS189	PMS190	atatGAATTCggtctcaggagGATCATGAGCGGAGAATTAAGGG	atatGGATCCggtctcacattATTGAGAGTGAATATGAGACTCTAATTG
pms5063	{P_MAS}	PMS191	PMS192	atatGAATTCggtctcaggagTTTTCAAATCAGTGCGCAAGAC	atatGGATCCggtctcacattCGATTTGGTGTATCGAGATTGG
pms5064	{T_tAtHsp}	PMS193	PMS194	atatGAATTCggtctcagcttATATGAAGATGAAGATGAAATATTTGGTGTG	atatGGATCCggtctcaagcgTTCCATAGTCCATACCATAGCAC
pms5065	{T_tAtRbcS}	PMS195	PMS196	atatGAATTCggtctcagcttCAGCACACTGGCGGC	atatAGATCTggtctcaagcgCAAGTCATAAAATGCATTAAAAAATATTTTCATAC
pms5066	{T_MAS}	PMS197	PMS198	atatGAATTCggtctcagcttCTTGGACTCCCATGTTGGC	atatGGATCCggtctcaagcgGATAATTTATTTGAAAATTCATAAGAAAAGCAAAC
pms5067	{L_tAtHsp}	PMS199	PMS200	atatGAATTCggtctcagtccATATGAAGATGAAGATGAAATATTTGGTGTG	atatGGATCCggtctcactccTTCCATAGTCCATACCATAGCAC
pms5068	{L_tAtRbcS}	PMS201	PMS202	atatGAATTCggtctcagtccCAGCACACTGGCGGC	atatAGATCTggtctcactccCAAGTCATAAAATGCATTAAAAAATATTTTCATAC
pms5069	{L_MAS}	PMS203	PMS204	atatGAATTCggtctcagtccCTTGGACTCCCATGTTGGC	atatGGATCCggtctcactccGATAATTTATTTGAAAATTCATAAGAAAAGCAAAC
pms5070	{C_GFP}	PMS205	PMS206	atatGAATTCggtctcaaatgGTGAGCAAGGGCGAGG	atatGGATCCggtctcaaagcTTACTTGTACAGCTCGTCCATG
pms5071	{C_GFP_attEpitope}	PMS207	PMS208	atatGAATTCggtctcaaatgGTGAGCAAGGGCGAGGAG	atatGGATCCggtctcaaagcCTAGAGCTCAGCGCTGTTATC
pms5072	{C_sfGFP}	PMS209	PMS210	atatGAATTCggtctcaaatgCGTAAAGGCGAAGAGCTGTTC	atatGGATCCggtctcaaagcTCATTTGTACAGTTCATCCATACCATG
pms5077	{P_AtUbq10}	PMS215	PMS249	atatGAATTCggtctcaggagTACCCGACGAGTCAGTAATAAAC	atatGGATCCggtctcacattAGTGTTAATCAGAAAAACTCAGATTAATCTAC
pms5078	{P_AtHsp18}	PMS216	PMS250	atatGAATTCggtctcaggagAAGCTTTTCTCTTCATTTCTCTTTCTTTC	atatGGATCCggtctcacattGACTTTTGGTTCTTGATTTTCCTGC
pms5079	{P_AtHsp70}	PMS217	PMS251	atatGAATTCggtctcaggagGAACTGCGAAAAAAGGGAGC	atatGGATCCggtctcacattTGCTAAAAAAAAGCTTCAGTAATTGAATG
pms5080	{P_AtAct2}	PMS218	PMS252	atatGAATTCggtctcaggagTCGACAAAATTTAGAACGAACTTAATTATG	atatGGATCCggtctcacattTTCAAAGCGGAGAGGAAAATATATG
pms5081	{P_At2S3}	PMS219	PMS253	atatGAATTCggtctcaggagGAAACCAAATTAACATAGGGTTTTTATTTAAATAAAAG	atatGGATCCggtctcacattGTTTTGCTATTTGTGTATGTTTTCTTG
pms5082	{P_AtBch1}	PMS220	PMS254	atatGAATTCggtctcaggagTGCCTTAATCTTGTCTGCAATG	atatGGATCCggtctcacattCCTAATGGAAGGAGGAGCTAC
pms5083	{P_AtUbq3}	PMS221	PMS255	atatCAATTGggtctcaggagCCGTACAAAATTGATTATTGATGTCACAAATC	atatGGATCCggtctcacattCTGAAATAAAACAATAGAACAAGTAGAAACC
pms5084	{P_AtPYK10}	PMS222	PMS256	atatGAATTCggtctcaggagATCTTTAAAAACCATTCTTTTATACCAAGCATG	atatGGATCCggtctcacattTTTTGTTTGTAATTCTGATTTTATTCAAGAAAAAATG
pms5085	{P_AtJAL34}	PMS223	PMS257	atatGAATTCggtctcaggagGCTAATGACATTAGATCGTAAATAAGTTCAGG	atatGGATCCggtctcacattCGTTGTTGACTTCTTTTATCTATCTGTG
pms5086	{P_AtWRKY6}	PMS224	PMS258	atatGAATTCggtctcaggagGTCGGTGACAAGCAACGAAAC	atatGGATCCggtctcacattATATAGAAAAAGAAAGAGATCACGAGATTAACG
pms5087	{P_At3g24240}	PMS225	PMS259	atatCAATTGggtctcaggagGAAAAAGTGATGGAAACTCGGAAAG	atatGGATCCggtctcacattTTGCCTCAAAATACGAAAAGAAGATTAAAATC
pms5088	{P_At2g45430}	PMS226	PMS260	atatGAATTCggtctcaggagAAACTGGTGCAACAGCTTTC	atatGGATCCggtctcacattTACTTGATATAAAAAGCAAACAAAAAGTAAAC
pms5089	{P_AtHY5}	PMS227	PMS261	atatGAATTCggtctcaggagGAGCATTGGGTGTTGGTG	atatTCTAGAggtctcacattTTTTCTTACTCTTTGAAGATCGATCAG
pms5090	{T_AtAct2}	PMS228	PMS262	atatGAATTCggtctcagcttGCTCTCAAGATCAAAGGCTTAAAAAG	atatGGATCCggtctcaagcgACTTTTTGGAATCAAGATGTATAAACCAAATG
pms5091	{T_AtBch1}	PMS229	PMS263	atatGAATTCggtctcagcttCTTTAAACAAGTTTTAAATCCCAAATTCTTTTTTTG	atatGGATCCggtctcaagcgAAGAGGAAAATTCCATAAACATCAATCTATG
pms5092	{T_AtHsp18.2}	PMS230	PMS264	atatGAATTCggtctcagcttATATGAAGATGAAGATGAAATATTTGGTGTG	atatGGATCCggtctcaagcgCTTATCTTTAATCATATTCCATAGTCCATACC
pms5093	{T_AtUbq3}	PMS231	PMS265	atatGAATTCggtctcagcttTAAGCTTTTTGTGATCTGATGATAAGTG	atatGGATCCggtctcaagcgTGTGGTAATCACACTATTGCACAC
pms5094	{T_AtPYK10}	PMS232	PMS266	atatGAATTCggtctcagcttGCTATTTCTGTTTCAATGTGTTTTTCC	atatGGATCCggtctcaagcgGCTGCGATGCAATCACTATGG
pms5095	{T_AtJAL34}	PMS233	PMS267	atatCAATTGggtctcagcttGTCGGTGACAAGCAACGAAAC	atatGGATCCggtctcaagcgGGGAGGAACTGAATTCGTACTGG
pms5096	{T_AtWRKY6}	PMS234	PMS268	atatGAATTCggtctcagcttAAAAATATTACATTTTTTTTTTGGGTATCTACAT	atatGGATCCggtctcaagcgCCATCTATTTACAATATTATTACTCCACAGTAC
pms5097	{T_At3g24240}	PMS235	PMS269	ATATAGAATTCcgtctcagcttTTTTAAGGTTTGAAGGTTACGTGAG	atatGGATCCcgtctcaagcgACAATTTACAAACTAAGGTATAGATGGAC
pms5098	{T_At2g45430}	PMS236	PMS270	atatCAATTGggtctcagcttATCGCGAAGAAAAAACAAGTTAGATAC	atatGGATCCggtctcaagcgTGCCTCTTGCCTCACCATC

pms5099	{T_AtHY5}	PMS237	PMS271	atatGAATTCggtctcagcttTCTCCTTCTTCTTCTTGTGTTATATTTT	atatGGATCCggtctcaagcgTCCATAGTACACTAGCAACTAGC
pms5100	{L_AtAct2}	PMS238	PMS272	atatGAATTCggtctcagtccGCTCTCAAGATCAAAGGCTTAAAAAG	atatGGATCCggtctcactccACTTTTTGGAATCAAGATGTATAAACCAAATG
pms5101	{L_AtBch1}	PMS239	PMS273	atatGAATTCggtctcagtccCTTTAAACAAGTTTTAAATCCCAAATTCTTTTTTTG	atatGGATCCggtctcactccAAGAGGAAAATTCCATAAACATCAATCTATG
pms5102	{L_AtHsp18.2}	PMS240	PMS274	atatGAATTCggtctcagtccATATGAAGATGAAGATGAAATATTTGGTGTG	atatGGATCCggtctcactccCTTATCTTTAATCATATTCCATAGTCCATACC
pms5103	{L_AtUbq3}	PMS241	PMS275	atatGAATTCggtctcagtccTAAGCTTTTTGTGATCTGATGATAAGTG	atatGGATCCggtctcactccTGTGGTAATCACACTATTGCACAC
pms5104	{L_AtPYK10}	PMS242	PMS276	atatGAATTCggtctcagtccGCTATTTCTGTTTCAATGTGTTTTTCC	atatGGATCCggtctcactccGCTGCGATGCAATCACTATGG
pms5105	{L_AtJAL34}	PMS243	PMS277	atatCAATTGggtctcagtccGTCGGTGACAAGCAACGAAAC	atatGGATCCggtctcactccGGGAGGAACTGAATTCGTACTGG
pms5106	{L_AtWRKY6}	PMS244	PMS278	atatGAATTCggtctcagtccAAAAATATTACATTTTTTTTTTGGGTATCTACAT	atatGGATCCggtctcactccCCATCTATTTACAATATTATTACTCCACAGTAC
pms5107	{L_At3g24240}	PMS245	PMS279	ATATAGAATTCcgtctcagtccTTTTAAGGTTTGAAGGTTACGTGAG	atatGGATCCcgtctcactccACAATTTACAAACTAAGGTATAGATGGAC
pms5108	{L_At2g45430}	PMS246	PMS280	atatCAATTGggtctcagtccATCGCGAAGAAAAAACAAGTTAGATAC	atatGGATCCggtctcactccTGCCTCTTGCCTCACCATC
pms5109	{L_AtHY5}	PMS247	PMS281	atatGAATTCggtctcagtccTCTCCTTCTTCTTCTTGTGTTATATTTT	atatGGATCCggtctcactccTCCATAGTACACTAGCAACTAGC
pms5125	{P_Casp1}	PMS328	PMS329n	atatGAATTCggtctcaggagAATAACGCATGCATTGTATTTGTATTC	atatAGATCTggtctcacattTTCTCTTGCAATTGGGGTTTAAAAG
pms5126	{P_DAISY}	PMS330n	PMS331	atatTCTAGAggtctcaggagATTAAATGCTCGCTTTGATTGATGC	atatGGATCCggtctcacattGGTAGGTTTTTTGGTTTTAAATGATAATCAAAAG
pms5127	{P_KCR1}	PMS332n	PMS333	atatTCTAGAggtctcaggagCCAAGAAAGTTGGAAGAGGAAG	atatGGATCCggtctcacattAGAGAAGAAAGGTTGAGACTTTGG
pms5128	{P_HORST}	PMS334n	PMS335n	atatTCTAGAcgtctcaggagTATGCATCGATGATGATTCTGCC	atatGGATCCcgtctcacattATCCCGGTTTAGGCTTTTTGC
pms5129	{P_CYP86B1}	PMS336n	PMS337n	atatGAATTCcgtctcaggagCTAAACACAACAAGTTTTCATGAGC	atatGGATCCcgtctcacattGTGACAAAGAGAAGAGAGAGCG
pms5130	{P_CYP705A1}	PMS338n	PMS339n	atatGAATTCcgtctcaggagGAAGAAATATACTCCTTTAGCAGGAAAC	atatGGATCCcgtctcacattGTTGCTGAAAAGCAAAGAAGAGG
pms5131	{P_GPAT5}	PMS340	PMS341n	atatGAATTCggtctcaggagTGATCGCAAACGTCAATGGTC	atatTCTAGAggtctcacattTCTTTTGTTTTTTGCTCGAATATTATTTTG
pms5132	{P_ASFT}	PMS342	PMS343	atatGAATTCggtctcaggagCAGGGTTCTTCATCATCATTCAC	atatGGATCCggtctcacattTTGATCCAAATGGAGAAAACAGC
pms5133	{P_FAR4}	PMS344	PMS345	atatGAATTCggtctcaggagAGTTCTAACCCCGACATAAGG	atatGGATCCggtctcacattGAAGAAACTTATATCTATCCAATTAATAACTAAGTAC
pms5793	{T_tG7}	PMS952	PMS953	tatatGAATTCggtctcagcttACTGACTAACTAGGATGAGCTAAGC	tatatGGATCCggtctcaagcgCTCGAGAAGCTTGGTACCCG
pms5794	{L_tG7}	PMS954	PMS955	atatGAATTCggtctcagtccACTGACTAACTAGGATGAGCTAAGC	atatGGATCCggtctcactccCTCGAGAAGCTTGGTACCCG
pms5124	{C_dsRed}	PMS752	PMS753	tatatGAATTCcgtctcaaatggcctcctccgagaacgtc	tatatGAATTCcgtctcaaagcctacaggaacaggtggtggc
pms5460	{C_GUS}	PMS946	PMS947n	tatatGAATTCggtctcaaatgGTCCGTCCTGTAGAAACCCCAACCC	tatatGGATCCggtctcaaagcTTATTGTTTGCCTCCCTGCTGCGGTTTTTC
pms5792	{P_SlE8}	PMS950	PMS951	ATATATACGTCTCAAATTggtctcaggagCATCCCTAGTGATATTGTTCACGTAATTAAG	ATATATACGTCTCAGATCggtctcacattCTTCTTTTGCACTGTGAATGATTAGAATAAT
pms5841	{P_OsHPX1}	PMS1004	PMS1005	atatGAATTCggtctcaggagACCTGCATATAGATGGGAAGTTCACTG	atatGGATCCggtctcacattGTTTCCCCCAAAAGCTTATGTGACC
pms5842	{P_TobRB7}	PMS1006	PMS1007	atatGAATTCggtctcaggagAAACACCCGAAAGGAAATGATTCGTTC	atatAGATCTggtctcacattGCGGATCCTAGTTCTCACTAGAAAAATG
pms5878	{P_ZmUBQ1}	PMS1051	PMS1052	atatataCGTCTCAAATTataggtctcaggagCAGTGCAGCGTGACCCG	atatataCGTCTCAGATCataggtctcacattCTAGAGTCGACCTGCAGAAGT
pms6116	{P_AtIrx5}	PMS1195	PMS1196	CGCTAAGGATGATTTCTGGAATTCggtctcaggagATGAAGCCATCCTCTACCTCGG	CTGCAGCTCGAGTTAGGATCCggtctcacattGGCGAGGTACACTGAGCTC
pms6117	{P_AtIrx8}	PMS1197	PMS1198	CGCTAAGGATGATTTCTGGAATTCcgtctcaggagACGAGCTGACTTGTACCGATG	CTGCAGCTCGAGTTAGGATCCcgtctcacattCGAAGAGGGAAACTGGATCTTA
pms6126	{P_AtCesA7}	PMS1199	PMS1200	CGCTAAGGATGATTTCTGGAATTCgaagacaaggagTGGGAACTTTCGGTACATTTTCC	CTGCAGCTCGAGTTAGGATCCgaagacaacattAGGGACGGCCGGAGATTAG
pms6392	{P_AtNST1}	pms6392F	pms6392R	atatataCAATTGggtctcAGGAGGTTTGTAGAGTTGGATCAG	atatataGGATCCggtctcacattCAAAGAGATTCAAGATTAACTCAAAAATCAAG
pms6393	{P_AtSAG12}	pms6393F	pms6393R	atatataCAATTGggtctcAGGAGGATTGTGATTTAATTTGTTGACTAG	atatataGGATCCggtctcacattGTTTTAGGAAAGTTAAATGACTTTTGCTCTT
pms6394	{P_AtVND6}	pms6394F	pms6394R	CGCTAAGGATGATTTCTGGAATTCggtctcAGGAGCTGTAGTTCACTTCTTTTTTCC	AGCTCGAGTTAGGATCCggtctcacattTGATCTTTTTAATTTTATATTTTCTGCAATGT
pms6395	{P_AtVND7}	pms6395F	pms6395R	atatataGAATTCggtctcAGGAGGGAATATATTTCTGTAGTTCTTC	atatataGGATCCggtctcacattCCACGATGATCCTATAAACGTATTTAATTTG
pms6396	{T_AtNST1}	pms6396F	pms6396R	atatataGAATTCcgtctcAGCTTCGGAAATGGAGGGAAAGAG	atatataGGATCCcgtctcaagcgGTGAGACATCAAACAACACCCCG
pms6397	{T_AtSAG12}	pms6397F	pms6397R	atatataGAATTCggtctcAGCTTATTCAAAACCGTGTTAAGCTTTTAATT	atatataGGATCCggtctcaagcgGAGAGTCAATGAATGTGAGAAAGAAGA
pms6398	{T_AtVND6}	pms6398F	pms6398R	atatataGAATTCggtctcAGCTTGATCAATCCTCAAAACGTGTG	atatataGGATCCggtctcaagcgGTTTGTGACTCAGGGGCTGTTTT
pms6399	{T_AtVND7}	pms6399F	pms6399R	atatataGAATTCggtctcAGCTTTTAAAAAAAAACACACTTCTATATATTGATTTG	atatataGGATCCggtctcaagcgAGGTTTTGACTTGGACTTCTTAATTGAAAC
pms6400	{L_AtNST1}	pms6400F	pms6400R	atatataGAATTCcgtctcAGTCCCGGAAATGGAGGGAAAG	atatataGGATCCcgtctcactccGTGAGACATCAAACAACACCCCG
pms6401	{L_AtSAG12}	pms6401F	pms6401R	atatataGAATTCggtctcAGTCCATTCAAAACCGTGTTAAGCTTTT	atatataGGATCCggtctcactccGAGAGTCAATGAATGTGAGAAAGAAGA
pms6402	{L_AtVND6}	pms6402F	pms6402R	atatataGAATTCggtctcAGTCCGATCAATCCTCAAAACGTG	atatataGGATCCggtctcactccGTTTGTGACTCAGGGGCTGTTTT
pms6403	{L_AtVND7}	pms6403F	pms6403R	atatataGAATTCggtctcAGTCCTTAAAAAAAAACACACTTCTATATATTGATT	atatataGGATCCggtctcactccAGGTTTTGACTTGGACTTCTTAATTGAAAC

pms6404	{T_AtHsp70}	pms6404F	pms6404R	atatataGAATTCggtctcAGCTTGCCTTTTGGCTTTTGTTTACTC	atatataGGATCCggtctcaagcgGCGCATAACTTCTTAAAAGAATTTTATTGTTC
pms6405	{T_At2S3}	pms6405F	pms6405R	atatataGAATTCggtctcAGCTTAGTCCAAGCCCGGCC	atatataGGATCCggtctcaagcgGTGTCCGGATCAGCCTGAC
pms6406	{T_AtFBA2}	pms6406F	pms6406R	atatataGAATTCcgtctcAGCTTAGAGATGATGCTGTGAAAAAAGA	atatataGGATCCcgtctcaagcgCGTTGCTGGTGTAACCGAGC
pms6408	{T_AtCASP1}	pms6408F	pms6408R	atatataGAATTCggtctcAGCTTTTTCATCTTTAAGTGTCAAGATCG	atatataGGATCCggtctcaagcgGGTCACGTGCGGTGAGGAG
pms6409	{T_AtKCR1}	pms6409F	pms6409R	atatataGAATTCggtctcAGCTTATCTTCCAGGTTTAAGTTACTAC	atatataGGATCCggtctcaagcgGCCGTTGGGTAAATAATGCGGG
pms6410	{T_AtASFT}	pms6410F	pms6410R	atatataGAATTCggtctcAGCTTTATGAACAACGACAAAATACAAATATC	atatataGGATCCggtctcaagcgCTACAACGGAGCAGGTTGTGTTT
pms6411	{L_AtHsp70}	pms6411F	pms6411R	atatataGAATTCggtctcAGTCCGCCTTTTGGCTTTTGTTTAC	atatataGGATCCggtctcactccGCGCATAACTTCTTAAAAGAATTTTATTGTTC
pms6412	{L_At2S3}	pms6412F	pms6412R	atatataGAATTCggtctcAGTCCAGTCCAAGCCCGGC	atatataGGATCCggtctcactccGTGTCCGGATCAGCCTGAC
pms6413	{L_AtFBA2}	pms6413F	pms6413R	atatataGAATTCcgtctcAGTCCAGAGATGATGCTGTGAAAAAAG	atatataGGATCCcgtctcactccCGTTGCTGGTGTAACCGAGC
pms6414	{L_AtUbq10}	pms6414F	pms6414R	atatataGAATTCggtctcAGTCCATCTCGTCTCTGTTATGCT	atatataGGATCCggtctcactccCACTTATGTAACATACACTTTCACTTGTG
pms6416	{L_AtCASP1}	pms6416F	pms6416R	atatataGAATTCggtctcAGTCCTTTCATCTTTAAGTGTCAAGATC	atatataGGATCCggtctcactccGGTCACGTGCGGTGAGGAG
pms6417	{L_AtKCR1}	pms6417F	pms6417R	atatataGAATTCggtctcAGTCCATCTTCCAGGTTTAAGTTACTAC	atatataGGATCCggtctcactccGCCGTTGGGTAAATAATGCGGG
pms6418	{L_AtASFT}	pms6418F	pms6418R	atatataGAATTCggtctcAGTCCTATGAACAACGACAAAATACAAATATC	atatataGGATCCggtctcactccCTACAACGGAGCAGGTTGTGTTT
pms6422	{P_AtAMT1.1}	pms6422F	pms6422R	AGGATGATTTCTGGAATTCggtctcAggagGAAATTTTATATCCTCTTCGATTTCTTTAC	AGCTCGAGTTAGGATCCggtctcAcattGTTGAGAGTTTAGAGAAGAAGATGAAAC
pms6424	{P_AtPht1.1}	pms6424F	pms6424R	CGCTAAGGATGATTTCTGGAATTCggtctcAggagGAGCCGCAAGAGATGACCAAG	AGCTCGAGTTAGGATCCggtctcAcattTCCTAGAGCTCTATAATCATACAACGC
pms6425	{P_AtPht1.4}	pms6425F	pms6425R	CGCTAAGGATGATTTCTGGAATTCggtctcAggagGAATAAACGTAGGTAGTGGCTACTT	AGCTCGAGTTAGGATCCggtctcAcattCTTCTTCTCCTCTGCAATTTTTCATCA
pms6426	{P_AtIRT1.1}	pms6426F	pms6426R	CGCTAAGGATGATTTCTGGAATTCggtctcAggagTTAGGAGCACATGGATTGACACATT	AGCTCGAGTTAGGATCCggtctcAcattTTTTTTTTTTTTCTTTTTTCTTTTGGATTGTT
pms6433	{T_AtIRT1.2}	pms6433F	pms6433R	atatataGAATTCggtctcAgcttACACTCTTTCAACATAATCAATAAATTATTTGATTT	atatataGGATCCggtctcAagcgGGTTAAATAGTTGTCATGAAATATTGTGTAATTTA
pms6434	{L_AtAMT1.1}	pms6434F	pms6434R	atatataGAATTCggtctcAgtccTTTGGATTTTTACTTTTATTCTCTATTTTCTAGAG	atatataGGATCCggtctcActccTGCTTCTAGAAAGCAATACTTCGAGTT
pms6435	{L_AtAMT1.3}	pms6435F	pms6435R	atatataGAATTCggtctcAgtccTTTTCAACTTTTTGGTAATTTATTACCGTTTAAGT	atatataGGATCCggtctcActccCGCTTTACGTGTACATAAGGGAATG
pms6436	{L_AtPht1.1}	pms6436F	pms6436R	atatataGAATTCggtctcAgtccTTATGTATGTTTATTTTGTTATTTGGAGTGCG	atatataGGATCCggtctcActccGGAAGCTTTTATTATGGTGTAGAAGTC
pms6437	{L_AtPht1.4}	pms6437F	pms6437R	atatataGAATTCggtctcAgtccGTGATATAATACGCCTTTTGTAATAATTTTCG	atatataGGATCCggtctcActccAACTTCCCCGCAACGCATAAATTG
pms6438	{L_AtIRT1.1}	pms6438F	pms6438R	atatataGAATTCggtctcAgtccCTAATACTCCAGATATTGCGGAATTGA	atatataGGATCCggtctcActccGGGACGATATGCAAGTGATTGAC
pms6439	{L_AtIRT1.2}	pms6439F	pms6439R	atatataGAATTCggtctcAgtccACACTCTTTCAACATAATCAATAAATTATTTGATTT	atatataGGATCCggtctcActccGGTTAAATAGTTGTCATGAAATATTGTGTAATTTA

Supplementary Table 7. Primers for assembly of R gene cluster library

Name	Primer Name	Sequence
R1-pt1F	PMS1502	CGTCTCGCATATCTCATTAAAGCAGGAACACGATTGCATAAAGTTTAGGTC
R1-pt1R	PMS1588	GTTCCTCTATGTAGAAGCAAAAATACATATAAG
R1-pt2F	PMS1604	CCTCCTTGAGAGTTGAGACC
R1-pt2R	PMS1605	GGACCATTTCAACATAGACCTTC
R1-pt3F	PMS1596	GGGAGGTCGGTTTCATGTCG
R1-pt3R	PMS1558	CTTTATTGCCAAATGTTTGAACGAAGCGCACGACAAATTCAAAACTAGAATATAGA C
R2-pt1F	PMS1503	CGTCTCGCATATCTCATTAAAGCAGCATGGGCTTTTTACTCTGATTTCAG
R2-pt1R	PMS1589	CGGAGTTTGTTTGGAAACAAATCG
R2-pt2F	PMS1597	GATTGTTTAGGCGCGTTTTTCTTG
R2-pt2R	PMS1559	CTTTATTGCCAAATGTTTGAACGAAGCGTATCATTTATACGAACACATTTAGTTTTG TAC
R3-pt1F	PMS1504	CGTCTCGCATATCTCATTAAAGCAGCAGATACCTCCCTCATATCCAAATAG
R3-pt1R	PMS1590	CAAGGCTTGTGGTTTGGTTT
R3-pt2F	PMS1598	CGATCCTTAAAATTAACAAACGAAGAATG
R3-pt2R	PMS1560	CTTTATTGCCAAATGTTTGAACGAAGCTTTGAGATTTACATGTGAGGTTTATGC
R4-pt1F	PMS1505	CGTCTCGCATATCTCATTAAAGCAGGGGGTCCTACGAAATGTCAAAATC
R4-pt1R	PMS1591	CAAGGGAGCTTCCACCATT
R4-pt2F	PMS1606	GCGTCTGCTTGAAGACTTTCAAAAATG
R4-pt2R	PMS1607	GGGTTTTTGAGAGATAATGCACTAGATG
R4-pt3F	PMS1599	CTTCGAACCGGAAAGTAATGGGTAG
R4-pt3R	PMS1561	CTTTATTGCCAAATGTTTGAACGAAGCGGCGAAAGACGACGGCTTAG
R5-pt1F	PMS1506	CGTCTCGCATATCTCATTAAAGCAGGAGAAACTGCTGGCCTAAGG
R5-pt1R	PMS1592	CGATGATGTCTCGCGTTGAAATC
R5-pt2F	PMS1600	GAGTACGATTGCAAAAGAGAAAAAAGAC
R5-pt2R	PMS1562	CTTTATTGCCAAATGTTTGAACGAAGCGGATATGTGTTAGACCCACTGACC
R6-pt1F	PMS1507	CGTCTCGCATATCTCATTAAAGCAGCAGTTTTATCACAGCAAGAATTCATTCC
R6-pt1R	PMS1593	GCACCTAAACATTCAATCGAATCTTTTC
R6-pt2F	PMS1608	GAAGGTTTCCCAGAATATGCC
R6-pt2R	PMS1609	GGCTTTAGTGCTCTCAAAACTGAAAG
R6-pt3F	PMS1601	GTCTTCTGTCTCACTATTCATCCG
R6-pt3R	PMS1563	CTTTATTGCCAAATGTTTGAACGAAGCAATTGCTTTTTTCCTTTTGGGTCTC
R7-pt1F	PMS1508	CGTCTCGCATATCTCATTAAAGCAGCTCTAAAGGTAGAAGCTTTTGGTCGG
R7-pt1R	PMS1594	GACAAGGCAGACAATTTGCAAG
R7-pt2F	PMS1602	GAAACTGTGCGTCTGGGTCG
R7-pt2R	PMS1564	CTTTATTGCCAAATGTTTGAACGAAGCAAAGACTAGAGCCATAGCCGA
R8-pt1F	PMS1509	CGTCTCGCATATCTCATTAAAGCAGAAAGCCTGAAGCTCAATTTTGATGG
R8-pt1R	PMS1595	GTGGAGGCCGGGAACCAG
R8-pt2F	PMS1610	CAAACATGTGGGTCCCATAAATG
R8-pt2R	PMS1611	CTTTTAGATTGGTGAGGAAAATCAATCC
R8-pt3F	PMS1603	GGGGGGGCTCTTAAGTCTTAAG

R8-pt3R	PMS1565	CTTTATTGCCAAATGTTTGAACGAAGCCATGTGATCTTGTAAATTAACTCGCGAG
R9-pt1F	PMS1423	GCATATCTCATTAAAGCAGGGAGCACATGAGAGAGATCTCTAGCTTAC
R9-pt1R	PMS1437	CAGTGAGCGAGGAAGCCCAAATGTAAAAAAACAACTGAGATTTGAGTG
R9-pt2F	PMS1424	GCTTTCGCTAAGGATGATTTCTGCTTTGAACTAACCTTTGATTTCGCCG
R9-pt2R	PMS1438	TGCCAAATGTTTGAACGAAGCACGAGACGATAGCACATATAGCAG
LinkerOcsF	PMS1465	CAACATAGTAAGCCAGTATACACTCCGAGGGGATCGAGCCCC
LinkerOcsR	PMS1466	CTGCTTTAATGAGATATGCGAGACG
TermNosF	PMS1566	GCTTCGTTCAAACATTTGGCAATAAAG
TermNosR	PMS1567	CCGGGCGTTATTTATTGGTGACCCGATCTAGTAACATAGATGACACC
SynCusterR2-pt1F	PMS1503	CGTCTCGCATATCTCATTAAAGCAGCATGGGCTTTTTACTCTGATTTCAG
SynCusterR2-pt1F	PMS1589	CGGAGTTTGTTTGGAAACAAATCG
SynCusterR2-pt2F	PMS1597	GATTGTTTAGGCGCGTTTTTCTTG
SynCusterR2-pt2F	PMS1559	CTTTATTGCCAAATGTTTGAACGAAGCGTATCATTTATACGAACACATTTAGTTTTG TAC
SynClusterLinkerF	PMS1546	CAACATAGTAAGCCAGTATACACTCCTTAATGGAGGAATATGATAGCTCAACAC
SynClusterLinkerR	PMS1547	GTATCATTTATACGAACACATTTAGTTTTGTAC
SynClusterR7-Pt1F	PMS1548	GTACAAAACTAAATGTGTTCGTATAAATGATACCTCTAAAGGTAGAAGCTTTTGGTC GG
SynClusterR7-Pt1R	PMS1549	CCGGGCGTTATTTATTGGTGAGACAAGGCAGACAATTTGCAAG
SynClusterR7-Pt2F	PMS1602	GAAACTGTGCGTCTGGGTCG
SynClusterR7-Pt2R	PMS1564	CTTTATTGCCAAATGTTTGAACGAAGCAAAGACTAGAGCCATAGCCGA

Supplemental References:

1 Farhi, M. *et al.* Generation of the potent anti-malarial drug artemisinin in tobacco. *Nat Biotech* **29**, 1072-1074 (2011).

2 Lu, W. *et al.* Identification and characterization of fructose 1,6-bisphosphate aldolase genes in Arabidopsis reveal a gene family with diverse responses to abiotic stresses. *Gene* **503**, 65-74, doi:http://dx.doi.org/10.1016/j.gene.2012.04.042 (2012).

3 Yang, F. *et al.* Engineering secondary cell wall deposition in plants. *Plant Biotechnol J* **11**, 325-335 (2013).

4 Langridge, W. H. R., Fitzgerald, K. J., Koncz, C., Schell, J. & Szalay, A. A. Dual promoter of Agrobacterium tumefaciens mannopine synthase genes is regulated by plant growth hormones. *Proc Natl Acad Sci* **86**, 3219-3223 (1989).

5 Sarrion-Perdigones, A. *et al.* GoldenBraid 2.0: A Comprehensive DNA Assembly Framework for Plant Synthetic Biology. *Plant Physiol* **162**, 1618-1631 (2013).

6 Lao, J. *et al.* The plant glycosyltransferase clone collection for functional genomics. *Plant J* **79**, 517-529 (2014).

7 Kittleson, J. T., Cheung, S. & Anderson, J. Rapid optimization of gene dosage in E. coli using DIAL strains. *J Biol Eng* **5**, 1-7 (2011).

8 Sung, D. Y., Vierling, E. & Guy, C. L. Comprehensive Expression Profile Analysis of the Arabidopsis Hsp70 Gene Family. *Plant Physiol* **126**, 789-800 (2001).

9 Emami, S., Yee, M.-c. & Dinneny, J. A robust family of Golden Gate Agrobacterium vectors for plant synthetic biology. *Front Plant Sci* **4**, 339 (2013).

10 Nitz, I., Berkefeld, H., Puzio, P. S. & Grundler, F. M. W. Pyk10, a seedling and root specific gene and promoter from Arabidopsis thaliana. *Plant Sci* **161**, 337-346 (2001).

11 Yamada, K., Hara-Nishimura, I. & Nishimura, M. Unique Defense Strategy by the Endoplasmic Reticulum Body in Plants. *Plant Cell Physiol* **52**, 2039-2049 (2011).

12 Robatzek, S. & Somssich, I. E. Targets of AtWRKY6 regulation during plant senescence and pathogen defense. *Genes Dev* **16**, 1139-1149 (2002).

13 Shinohara, H., Mori, A., Yasue, N., Sumida, K. & Matsubayashi, Y. Identification of three LRR-RKs involved in perception of root meristem growth factor in Arabidopsis. *Proc Natl Acad Sci* **113**, 3897-3902 (2016).

14 Zhao, J., Favero, D. S., Peng, H. & Neff, M. M. Arabidopsis thaliana AHL family modulates hypocotyl growth redundantly by interacting with each other via the PPC/DUF296 domain. *Proc Natl Acad Sci* **110**, E4688-E4697 (2013).

15 Chattopadhyay, S., Ang, L.-H., Puente, P., Deng, X.-W. & Wei, N. Arabidopsis bZIP Protein HY5 Directly Interacts with Light-Responsive Promoters in Mediating Light Control of Gene Expression. *Plant Cell* **10**, 673-683 (1998).

16 Naseer, S. *et al.* Casparian strip diffusion barrier in Arabidopsis is made of a lignin polymer without suberin. *Proc Natl Acad Sci* **109**, 10101-10106 (2012).

17 Butaye, K. M. J. *et al.* Stable high-level transgene expression in Arabidopsis thaliana using gene silencing mutants and matrix attachment regions. *Plant J* **39**, 440-449 (2004).

18 Wally, O., Jayaraj, J. & Punja, Z. K. Comparative expression of β-glucuronidase with five different promoters in transgenic carrot (Daucus carota L.) root and leaf tissues. *Plant Cell Rep* **27**, 279-287 (2008).

19 Butelli, E. *et al.* Enrichment of tomato fruit with health-promoting anthocyanins by expression of select transcription factors. *Nat Biotech* **26**, 1301-1308 (2008).

20 Park, S.-H. *et al.* Characterization of the root-predominant gene promoter HPX1 in transgenic rice plants. *Plant Biotechnol Rep* **7**, 339-344 (2013).

21 Yamamoto, Y. T., Taylor, C. G., Acedo, G. N., Cheng, C. L. & Conkling, M. A. Characterization of cis-acting sequences regulating root-specific gene expression in tobacco. *Plant Cell* **3**, 371-382 (1991).

22 Cornejo, M.-J., Luth, D., Blankenship, K. M., Anderson, O. D. & Blechl, A. E. Activity of a maize ubiquitin promoter in transgenic rice. *Plant Mol Biol* **23**, 567-581 (1993).

23 Gondolf, V. M. *et al.* A gene stacking approach leads to engineered plants with highly increased galactan levels in Arabidopsis. *BMC Plant Biol* **14**, 1-11, doi:10.1186/s12870-014-0344-x (2014).

24 Kim, W.-C. *et al.* MYB46 directly regulates the gene expression of secondary wall-associated cellulose synthases in Arabidopsis. *Plant J* **73**, 26-36 (2013).

25 Vega-Sánchez, M. E. *et al.* Engineering temporal accumulation of a low recalcitrance polysaccharide leads to increased C6 sugar content in plant cell walls. *Plant Biotechnology Journal* **13**, 903-914, doi:10.1111/pbi.12326 (2015).

26 Kubo, M. *et al.* Transcription switches for protoxylem and metaxylem vessel formation. *Genes Dev* **19**, 1855-1860 (2005).

27 Loqué, D. *et al.* Additive contribution of AMT1;1 and AMT1;3 to high-affinity ammonium uptake across the plasma membrane of nitrogen-deficient Arabidopsis roots. *Plant J* **48**, 522-534 (2006).

28 Mudge, S. R., Rae, A. L., Diatloff, E. & Smith, F. W. Expression analysis suggests novel roles for members of the Pht1 family of phosphate transporters in Arabidopsis. *Plant J* **31**, 341-353 (2002).

29 Vert, G. *et al.* IRT1, an Arabidopsis Transporter Essential for Iron Uptake from the Soil and for Plant Growth. *Plant Cell* **14**, 1223-1233 (2002).

30 Varotto, C. *et al.* The metal ion transporter IRT1 is necessary for iron homeostasis and efficient photosynthesis in Arabidopsis thaliana. *Plant J* **31**, 589-599 (2002).
